# Bibliometric analysis of the *Journal of Shoulder and Elbow Surgery*: citation trends, evidence levels, and scholarly impact

**DOI:** 10.1016/j.jseint.2026.101691

**Published:** 2026-03-06

**Authors:** Joseph Torkieh, Keanu Shah, Kira N. Kornienko, James M. Lee, Rahul Mittal

**Affiliations:** a Department of Orthopedics, Rutgers Robert Wood Johnson Medical School, New Brunswick, NJ, USA; b Modern Orthopedics of New Jersey, North Paramus, NJ, USA; c Department of Health Informatics, Rutgers - School of Health Professions, Piscataway, NJ, USA

**Keywords:** Bibliometrics, Citation analysis, Shoulder surgery, Evidence level, Authorship trend, Orthopedics

## Abstract

**Background:**

The *Journal of Shoulder and Elbow Surgery (JSES)* is a leading publication for upper-extremity research; however, no comprehensive bibliometric evaluation of its most influential work has been performed. This study aimed to identify the most-cited articles and authors in JSES and to analyze trends in evidence level, study design, and authorship characteristics.

**Methods:**

A bibliometric analysis using Scopus identified all *JSES* publications from 1992 to 2024. The 20 most-cited articles and 100 most-cited authors were assessed for citation impact, article type, evidence level, author demographics, and relative citation index. Authorship patterns were analyzed across first, last, and any author positions.

**Results:**

Among 7,171 *JSES* publications, 606 articles (8.5%) had ≥100 citations. The top 20 articles received 495–1,441 citations and were all clinical studies, most commonly Level II (55%) and Level IV (30%). The most-cited article was “A standardized method for the assessment of shoulder function” (1,441 citations). Gilles Walch (France) was the most-cited author overall (10,908 citations), while John W. Sperling (USA) was the most prolific (134 publications). U.S.-based authors represented the majority of highly cited contributors, followed by France and Switzerland, while female and non-Western authors remained underrepresented.

**Conclusion:**

Highly cited *JSES* publications are predominantly mid-level evidence of clinical studies, reflecting the practical nature of upper-extremity research and challenges in conducting randomized trials. Authorship trends show both increasing participation and broad international involvement. Improving study quality and continuing to broaden global representation are key priorities for future scholarship.

Bibliometric analysis provides a quantitative approach for evaluating the scholarly influence, authorship patterns, and methodological characteristics of research within a discipline. In orthopedic surgery, such analyses have been applied to subspecialties including shoulder instability, elbow surgery, reverse arthroplasty, and sports medicine, consistently demonstrating that the most influential literature is predominantly composed of Level III and IV studies.^1^^,^^2^^,^^9^^,^^13^^,^^16^^,^^27^^,^^36^ These reviews have helped define historical trends, highlight emerging research priorities, and identify areas where methodological rigor remains limited.

The *Journal of Shoulder and Elbow Surgery* (*JSES*) serves as the flagship publication venue for upper-extremity research and is widely recognized as one of the leading sources of influential scholarship in shoulder and elbow surgery.^9^^,^^16^^,^^27^^,^^36^ Since its inception in 1992, *JSES* has played a central role in shaping the scientific foundation of the specialty; however, no comprehensive bibliometric analysis has examined its most impactful publications, authors, or trends over time. A focused evaluation of *JSES* is needed to identify its foundational contributions, assess patterns in evidence levels and study design, and determine whether citation impact aligns with methodological quality and authorship diversity.

Accordingly, the purpose of this study was to (1) identify the most-cited *JSES* articles and authors; (2) evaluate study type, evidence level, and citation performance; and (3) analyze authorship characteristics, including gender, geographic distribution, and practice setting. We hypothesized that the most highly cited studies would predominantly consist of clinical research with Level II–IV evidence and that authorship would be disproportionately concentrated among North American and European institutions, with women and low- and middle-income countries underrepresented.

## Materials and methods

### IRB approval

This study was a retrospective bibliometric analysis involving only publicly available publication data and did not include human participants, identifiable private information, or animal subjects. In accordance with institutional and federal regulations, the study was considered nonhuman subjects research and therefore did not require institutional review board approval.

### Literature search

All articles published in the *JSES* between January 1992 and September 2025 were identified using the Scopus database. The database was queried in September 2025, at which time Scopus had already indexed articles assigned 2026 publication dates through early online publication and advance issue assignment; these articles were therefore included in the analysis. Scopus was selected because of its broad citation coverage, robust author disambiguation capabilities, and efficient export tools, which make it particularly well suited for bibliometric analyses. A journal-level query using the “Source Title” filter for *JSES* was performed, and all indexed records were retrieved without restrictions on article category or publication type.

### Eligibility criteria

All retrieved articles were screened to determine eligibility. Eligible studies included full-length clinical and basic science research articles such as original investigations, observational studies, cohort studies, biomechanical analyses, and cadaveric or anatomical research. Editorials, commentaries, letters, conference abstracts, errata, retractions, and non-indexed materials were excluded. Case reports were also excluded to ensure uniformity of study type and because they contribute minimally to comparative bibliometric analyses.

### Data extraction

For each eligible article, information was collected on title, publication year, total citations, annual citation rate, article type and subtype, and level of evidence. The complete author list was recorded, with specific attention to first-, last-, and any-author positions. Levels of Evidence were assigned using the Oxford Centre for Evidence-Based Medicine criteria. When a study did not explicitly report a level of evidence, it was independently determined by the authors based on Centre for Evidence-Based Medicine definitions, ensuring consistent application across all included studies.

### Article classification

Articles were categorized using established bibliometric methodology.^6^ Clinical investigations were classified as prospective cohort studies, retrospective cohort studies, case series, case-control studies, diagnostic accuracy studies, non-randomized comparative studies, or randomized controlled trials. Basic science manuscripts were classified as cadaveric or anatomical investigations, biomechanical studies, or laboratory-based experimental research. Systematic reviews were assigned the level of evidence corresponding to the lowest level among the primary studies they analyzed. All classifications and level-of-evidence determinations were applied uniformly across the dataset.

### Identification and demographics

To characterize scholarly contribution patterns within the journal, the top 100 most-cited authors were identified based on total citation counts in first-author, last-author, and any-author positions. Author identities were verified using Scopus Author IDs and confirmed by manual review of institutional profiles, professional biographies, and publicly accessible faculty listings. Demographic characteristics—including country of current practice, country of residency training, practice setting (academic or private), and sex—were documented when available. Sex was inferred from publicly available institutional or biographical sources.

### Relative citation index

To normalize citation performance across publication years, a relative citation index (RCI) was calculated for each article by dividing an article's total citation count by the mean citation count of all *JSES* articles published in the same year. Separate RCIs were computed for all publications combined as well as for first-author and last-author contributions.

$$RCI=\frac{Total\;Citations}{Total\;number\;of\;articles}$$

### Statistical analysis

This study used descriptive bibliometric methods. Continuous variables are reported as means and medians, while categorical variables are reported as frequencies and percentages. Univariate linear regression analysis was used to determine the association between publication year and the number of authors per manuscript.

## Results

Since the *JSES* began publication in 1992, a total of 7,171 articles have been published and were included in this analysis. Of these, 606 articles—representing 8.5% of all JSES publications—had accumulated at least 100 citations. Across the full dataset, the mean citation count was 38 citations per article, while the median was 19 citations, reflecting the expected right-skewed distribution of citation data.

### Top 20 *Journal of Shoulder and Elbow Surgery* cited articles

The 20 most-cited articles^3^, ^4^, ^5^^,^^7^^,^^10^, ^11^, ^12^^,^^15^^,^^18^^,^^20^^,^^22^^,^^25^^,^^26^^,^^28^, ^29^, ^30^, ^31^, ^32^^,^^34^^,^^35^ in *JSES* collectively received 15,029 citations (Table I). Individual citation counts ranged from 495 to 1,441, with mean and median values of 752 and 673, respectively. Annual citation rates ranged from 19 to 71 citations per year. The most-cited article, “A standardized method for the assessment of shoulder function,” published in 1994, accounted for 1,441 citations.^26^ All 20 articles were clinical studies and spanned evidence levels II through V, with Level II studies^3^^,^^7^^,^^10^, ^11^, ^12^^,^^15^^,^^18^^,^^20^^,^^25^^,^^29^^,^^35^ comprising the largest proportion, followed by level IV,^4^^,^^5^^,^^22^^,^^30^, ^31^, ^32^ III,^28^^,^^34^ and V.^26^

**Table I:** Top 20 articles in the *Journal of Shoulder and Elbow Surgery* and their characteristics.

Rank	Lead author	Title	Year published	Citations	Citations/year since publication	Article type	Subtype	Level of evidence	Subject
1	Richards et al^26^	A standardized method for the assessment of shoulder function	1994	1,441	46.5	Clinical	Descriptive study	V	Shoulder function assessment
2	Fuchs et al^10^	Fatty degeneration of the muscles of the rotator cuff: Assessment by computed tomography versus magnetic resonance imaging	1999	1,196	46.0	Clinical	Prospective cohort	II	Rotator cuff degeneration
3	Yamamoto et al^34^	Prevalence and risk factors of a rotator cuff tear in the general population	2010	1,057	70.5	Clinical	Population-based study	III	Rotator cuff tear prevalence and treatment
4	Boileau et al^5^	Neer Award 2005: The Grammont reverse shoulder prosthesis: Results in cuff tear arthritis, fracture sequelae, and revision arthroplasty	2006	957	50.4	Clinical	Retrospective cohort	IV	Reverse shoulder prosthesis functionality
5	Michener et al^20^	American Shoulder and Elbow Surgeons Standardized Shoulder Assessment Form, patient self-report section: Reliability, validity, and responsiveness	2002	904	39.3	Clinical	Prospective observational study	II	Shoulder function assessment
6	Goutallier et al^12^	Influence of cuff muscle fatty degeneration on anatomic and functional outcomes after simple suture of full-thickness tears	2003	814	37.0	Clinical	Prospective cohort	II	Impact of pre-operative fatty degeneration on long-term cuff integrity
7	Tempelhof et al^29^	Age-related prevalence of rotator cuff tears in asymptomatic shoulders	1999	730	28.1	Clinical	Prospective cross-sectional	II	Prevalence of asymptomatic rotator cuff tears increases with age
8	Day et al^7^	Prevalence and projections of total shoulder and elbow arthroplasty in the United States to 2015	2010	710	47.3	Clinical	Epidemiological projection study	II	Current and projected utilization rates of shoulder and elbow arthroplasty
9	Postacchini et al^25^	Epidemiology of clavicle fractures	2002	706	30.7	Clinical	Retrospective observational epidemiology	II	Rate and distribution of clavicle fractures
10	Gilbart et al^11^	Comparison of the subjective shoulder value and the Constant score	2007	687	38.2	Clinical	Prospective observational	II	Comparison of patient-reported SSV vs. Constant score in different shoulder pathologies
11	Nagels et al^22^	Stress shielding and bone resorption in shoulder arthroplasty	2003	659	30.0	Clinical	Retrospective radiographic review	IV	Radiographic assessment of stress shielding after humeral head replacements
12	Hertel et al^15^	Predictors of humeral head ischemia after intracapsular fracture of the proximal humerus	2004	625	29.8	Clinical	Prospective cohort	II	Identification of predictors of humeral head ischemia
13	Walch, Gilles et al^31^	Impingement of the deep surface of the supraspinatus tendon on the posterosuperior glenoid rim: An arthroscopic study	1992	610	18.5	Clinical	Arthroscopic case series	IV	Arthroscopic examination of supraspinatus–glenoid impingement in athletes
14	Yamamoto et al^35^	Contact between the glenoid and the humeral head in abduction, external rotation, and horizontal extension: A new concept of glenoid track	2007	606	33.7	Clinical	Cadaveric biomechanical study	II	Description of glenoid-track concept via cadaver analysis of glenoid-humeral contact
15	Torchia et al^30^	Total shoulder arthroplasty with the Neer prosthesis: Long-term results	1997	595	21.3	Clinical	Retrospective case series	IV	Long-term outcomes and survival rate of Neer total shoulder prosthesis
16	Boileau et al^4^	Tuberosity malposition and migration: Reasons for poor outcomes after hemiarthroplasty for displaced fractures of the proximal humerus	2002	580	25.2	Clinical	Retrospective case series	IV	Clinical and radiographic evaluation of tuberosity positioning after hemiarthroplasty for proximal humerus fracture
17	Walch et al^32^	Arthroscopic tenotomy of the long head of the biceps in the treatment of rotator cuff tears: Clinical and radiographic results of 307 cases	2005	555	27.8	Clinical	Retrospective consecutive case series	IV	Clinical and radiographic outcomes following arthroscopic biceps tenotomy in rotator cuff tear patients
18	McClure et al^18^	Direct 3-dimensional measurement of scapular kinematics during dynamic movements in vivo	2001	553	23.0	Clinical	Experimental biomechanics	II	Describes in vivo scapular motion during dynamic shoulder movements using bone-pin–mounted sensors
19	Tashjian et al^28^	Minimal clinically important differences (MCID) and patient acceptable symptomatic state (PASS) for visual analog scales (VAS) measuring pain in patients treated for rotator cuff disease	2009	549	34.3	Clinical	Prospective diagnostic	III	Defines thresholds for clinically meaningful improvement and acceptable state on 10 cm VAS in nonoperative rotator cuff patients
20	Bishop et al^3^	Cuff integrity after arthroscopic versus open rotator cuff repair: A prospective study	2006	495	26.1	Clinical	Comparative study	II	Comparison of strategies for rotator cuff repair

*SSV*, Subjective Shoulder Value.

### Top 20 *Journal of Shoulder and Elbow Surgery* cited any position author

Analysis of authorship impact revealed similar patterns of concentrated scholarly influence. The top 20 authors (Table II) ranked by total citations in any authorship position had accumulated 109,990 citations. Citation counts in this group ranged from 3,095 to 10,908, and authors contributed between 27 and 134 publications each. Within this cohort, Gilles Walch had the highest citation total, whereas John W. Sperling was the most prolific contributor, and Kainan An had the highest RCI.

**Table II:** Top 20 authors in the *Journal of Shoulder and Elbow Surgery* ranked by total citations as an author in any position.

Rank	Authors	Current institutions	Country	No. of citations	No. of articles published	Total RCI
1	Walch, Gilles	Hopital Privé J Mermoz Ramsay Générale de Santé	France	10,908	95	40.6
2	Gerber, Christian	Uniklinik Balgrist	Switzerland	8,680	81	114.8
3	Cofield, Robert	Mayo Clinic	United States	8,412	122	107.2
4	Boileau, Pascal	ICR-Institut de Chirurgie Réparatrice Locomoteur & Sport-Groupe KANTYS	France	7,762	66	69.0
5	Sperling, John	Mayo Clinic	United States	7,191	134	117.6
6	Zuckerman, Joseph	NYU Grossman School of Medicine	United States	7,140	96	53.7
7	Iannotti, Joseph	Cleveland Clinic Lerner College of Medicine of Case Western Reserve University	United States	6,503	73	74.4
8	Warren, Russell Frederick	Hospital for Special Surgery - New York	United States	5,643	78	89.1
9	Bigliani, Louis	Columbia University Irving Medical Center	United States	5,280	45	72.3
10	Williams, Gerald Ross	Thomas Jefferson University Hospital	United States	4,726	71	117.3
11	Frankle, Mark	Florida Orthopaedic Institute	United States	4,327	77	66.6
12	Warner, Jon	Massachusetts General Hospital	United States	4,285	64	56.2
13	Romeo, Anthony	Rush University Medical Center	United States	4,257	86	67.0
14	Flatow, Evan	Icahn School of Medicine at Mount Sinai	United States	3,748	48	49.5
15	Edwards, Thomas Bradley	Baylor St. Luke's Medical Center	United States	3,717	48	78.1
16	Matsen III, Frederick	University of Washington	United States	3,674	80	77.4
17	Wright, Thomas	University of Florida	United States	3,645	101	45.9
18	Gartsman, Gary	Texas Orthopedic Hospital	United States	3,643	27	36.1
19	An, Kainan	Mayo Clinic	United States	3,354	48	134.9
20	Sánchez-Sotelo, Joaquín	Mayo Clinic	United States	3,095	94	69.9

*RCI*, relative citation index.

### Top 100 *Journal of Shoulder and Elbow Surgery* cited any position author

Expanding this analysis to the top 100 most-cited authors showed a total of 265,956 citations (Supp Table 1), with citation counts ranging from 1,441 to 10,908 and publication counts ranging from 1 to 134. While Gilles Walch and John W. Sperling again led in citation and publication volumes, respectively, Anthony Gristina produced the highest RCI based on a single, highly cited article.

### Top 20 *Journal of Shoulder and Elbow Surgery* cited first and last authors

Examining first authorship provided additional insights into research leadership within the journal. The top 20 most-cited first authors collectively accumulated 27,328 citations (Table III), with individual citation totals ranging from 862 to 4,127. Within this group, Pascal Boileau led in both citation and publication volume, while Bruno Fuchs achieved the highest RCI. Fourteen of these first authors also published as last authors, and within this subgroup, Christian A. Gerber demonstrated the highest last-author citation count, publication count, and RCI (Table IV).

**Table III:** Top 20 authors in the *Journal of Shoulder and Elbow Surgery* ranked by total citations as the first author.

Rank	Author	Current institution	Country	No. of first author citations	No. of articles published as the first author	First author RCI
1	Boileau, Pascal	ICR-Institut de Chirurgie Réparatrice Locomoteur & Sport-Groupe KANTYS	France	4,127	24	172.0
2	Walch, Gilles	Hopital Privé J Mermoz Ramsay Générale de Santé	France	2,869	12	239.1
3	Tashjian, Robert Zaray	University of Utah School of Medicine	United States	1,571	19	82.7
4	Richards, Robin	University of Toronto	Canada	1,517	3	505.7
5	Hovelius, Lennart	Gavle Hospital (2016)	Sweden	1,512	7	216.0
6	Gerber, Christian	Uniklinik Balgrist	Switzerland	1,378	11	125.3
7	Hertel, Ralph	Schulter & amp; Ellbogen Zentrum Bern	Switzerland	1,341	8	167.6
8	Fuchs, Bruno	Kantonsspital Winterthur	Switzerland	1,323	2	661.5
9	Sperling, John	Mayo Clinic	United States	1,202	10	120.2
10	Yamamoto, Atsushi	Graduate School of Medicine	Japan	1,175	3	391.7
11	Warner, Jon	Massachusetts General Hospital	United States	1,042	12	86.8
12	Edwards, Thomas Bradley	Baylor St. Luke's Medical Center	United States	1,016	8	127.0
13	Michener, Lori Ann	University of Southern California	United States	979	2	489.5
14	Yamamoto, Nobuyuki	Tohoku University School of Medicine	Japan	940	9	104.4
15	Goutallier, Daniel	Hôpital Henri Mondor (2019)	France	932	3	310.7
16	Day, Judd	Drexel University	United States	896	6	149.3
17	Gartsman, Gary	Texas Orthopedic Hospital	United States	895	9	99.4
18	Nyffeler, Richard	Orthopädie Sonnenhof	Switzerland	884	4	221.0
19	Levy, Ofer	Berkshire Independent Hospital	United Kingdom	867	12	72.3
20	Postacchini, Franco	Sapienza Università di Roma	Italy	862	2	431.0

*RCI*, relative citation index.

**Table IV:** Top 20 authors in the *Journal of Shoulder and Elbow Surgery* ranked by total citations as the last author.

Rank	Author	Current institution	Country	No. of last author citations	No. of articles published as the last author	Last author RCI
1	Gerber, Christian	Uniklinik Balgrist	Switzerland	6,548	54	121.3
2	Walch, Gilles	Hopital Privé J Mermoz Ramsay Générale de Santé	France	5,261	47	111.9
3	Cofield, Robert	Mayo Clinic	United States	4,107	62	66.2
4	Matsen Iv, Frederick	University of Washington	United States	3,123	52	60.1
5	Iannotti, Joseph	Cleveland Clinic Lerner College of Medicine of Case Western Reserve University	United States	2,955	38	77.8
6	Lee, Thay	Congress Medical Foundation – Orthopaedic Biomechanics Laboratory	United States	2,839	51	55.7
7	Frankle, Mark	Florida Orthopaedic Institute	United States	2,684	56	47.9
8	Zuckerman, Joseph	NYU Grossman School of Medicine	United States	2,309	40	57.7
9	Warren, Russell Frederick	Hospital for Special Surgery - New York	United States	2,175	27	80.6
10	Flatow, Evan	Icahn School of Medicine at Mount Sinai	United States	2010	23	87.4
11	Warner, Jon	Massachusetts General Hospital	United States	1994	33	60.4
12	Hawkins, Richard	Steadman-Hawkins Clinic	United States	1734	22	78.8
13	Bigliani, Louis	Columbia University Irving Medical Center	United States	1,625	20	81.3
14	Athwal, George	Hand and Upper Limb Centre	Canada	1,606	55	29.2
15	O'Driscoll, Shawn	Mayo Clinic	United States	1,397	48	29.1
16	Rowland, Charles	Mayo Clinic in Rochester, Minnesota	United States	1,385	7	197.9
17	Murrell	UNSW Sydney	Australia	1,292	28	46.1
18	Edwards, Thomas Bradley	Baylor St. Luke's Medical Center	United States	1,234	21	58.8
19	Gulotta, Lawrence Vincent	Hospital for Special Surgery - New York	United States	1,167	21	55.6
20	Namdari, Surena	Rothman Orthopaedics	United States	1,157	44	26.3

*RCI*, relative citation index.

### Top 100 *Journal of Shoulder and Elbow Surgery* cited first and last authors

Among the broader cohort of the top 100 most-cited first authors (Supp Table 2)—who collectively received 69,451 citations—Pascal Boileau again led in citation count, while Siegbert Tempelhof achieved the highest RCI from a single publication. Many of these first authors were also active as last authors (Supp Table 3), with 73 contributing last-author publications. In this group, Christian A. Gerber had the highest last-author citation count, Mark A. Frankle published the most last-author papers, and Christophe Lévigne achieved the highest last-author RCI.

### Characteristics of the top 100 *Journal of Shoulder and Elbow Surgery* cited first authors

The characteristics of these top first authors further illuminate authorship demographics in upper-extremity research. Of the top 100 first authors, 74 held MD degrees, 14 held combined MD/PhD degrees, 1 held an MSE/MBA, and 4 had unverified degrees. Within the MD group, 1 author also held an MPH, and another held an MBA. Only 5 women appeared in the top 100 (Lori Ann Michener, April Dawn Armstrong, Julie Y. Bishop, Emilie V. Cheung, and Birgit S. Werner).

### Country of training of the top 100 *Journal of Shoulder and Elbow Surgery* cited first authors

**Figure 1:**
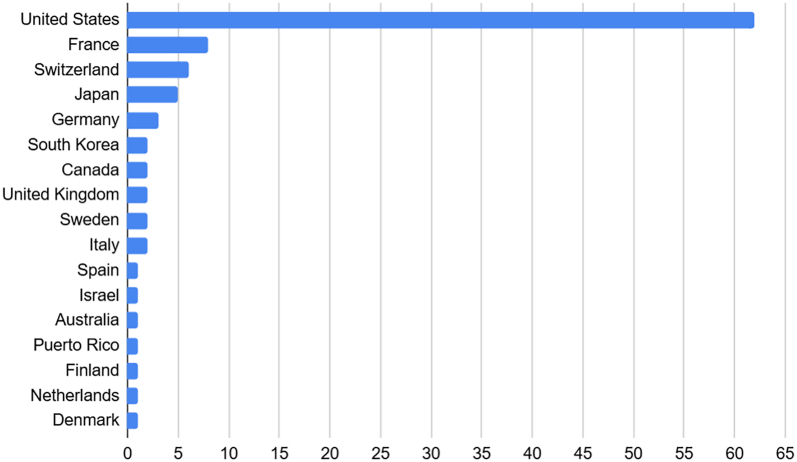
Current location of practice by country for authors with the top 100 first author citations.

Training and practice locations demonstrated a strong geographic concentration. Of the 82 first authors with known residency training, 52 (63%) trained in the United States, followed by France and Switzerland (each 7%), Canada (5%), Germany (4%), and Italy and the United Kingdom (each 2%) (Fig. 1).

### Current location of practice for the top 100 *Journal of Shoulder and Elbow Surgery* cited first authors

**Figure 2:**
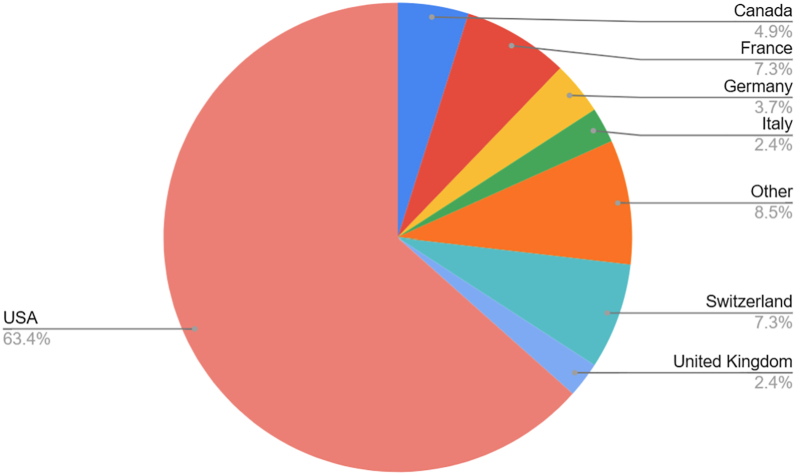
Country of training for MD authors within the top 100 *JSES* cited first authors (n = 81). “Other” includes 1 author from the following countries: Australia, Denmark, Israel, Japan, South Korea, Spain, and Sweden. *JSES, Journal of Shoulder and Elbow Surgery*.

**Figure 3:**
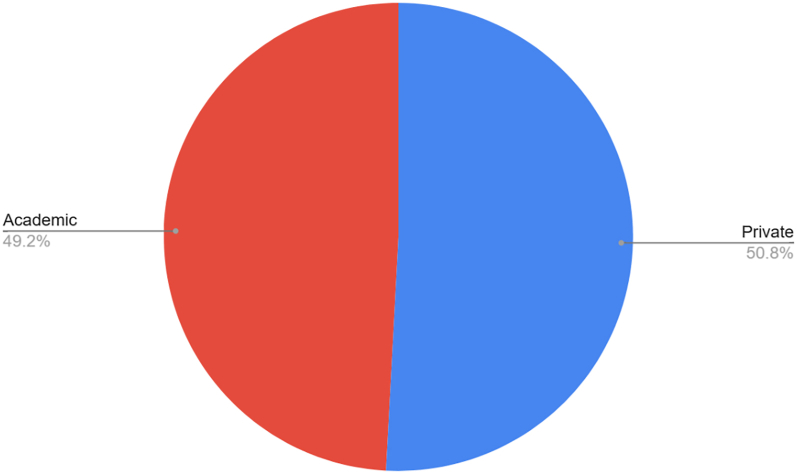
Current practice setting for the top 100 authors ranked by first author citations currently practicing in the United States.

Among current practice locations, 62 of the top 100 first authors were practicing in the United States, followed by France (n = 8), Switzerland (n = 6), Japan (n = 5), and Germany (n = 3) (Fig. 2). Among U.S.-based authors, practice environments were nearly evenly split between private practice (51%) and academic settings (49%) (Fig. 3).

### Manuscript authorship trend

Since *JSES*’ inception, the average number of authors per manuscript has steadily increased from a mean of 3.3 authors per manuscript in 1992 to 7.0 in 2026. Over this 35-year period, the number of authors per manuscript has increased at a statistically significant rate (*P* < .001, R^2^ = 0.94). A more in-depth understanding of this trend can be seen in Table V.

**Table V:** Average number of authors per manuscript from 1992 to 2026.

Year	Mean	Standard deviation
1992	3.3	1.5
1993	3.3	1.4
1994	3.3	1.6
1995	3.1	1.3
1996	3.7	1.5
1997	3.5	1.1
1998	3.6	1.4
1999	3.7	1.4
2000	3.5	1.4
2001	4.0	1.5
2002	4.1	1.5
2003	4.1	1.6
2004	3.9	1.6
2005	4.3	1.6
2006	4.1	1.6
2007	4.4	1.6
2008	4.5	1.7
2009	4.6	1.8
2010	4.6	1.7
2011	4.7	1.8
2012	4.9	1.9
2013	5.4	2.2
2014	5.4	2.3
2015	5.4	2.6
2016	5.3	2.1
2017	5.7	2.6
2018	5.6	2.2
2019	6.6	6.4
2020	6.1	3.8
2021	6.4	3.1
2022	6.7	3.0
2023	7.1	5.3
2024	7.2	5.0
2025	7.3	4.6
2026	6.9	2.4

## Discussion

This bibliometric analysis of the *JSES* identifies several notable trends in publication characteristics, study design, authorship patterns, and evidence levels over the past 3 decades. Consistent with findings from bibliometric analyses across other orthopedic subspecialties, the data demonstrate evolving patterns of citation impact, collaborative authorship, and methodological rigor within upper-extremity research.

One of the most notable findings of this study is the limited presence of Level I randomized controlled trials among the journal's most-cited publications. This pattern mirrors prior bibliometric evaluations in shoulder instability, rotator cuff pathology, elbow surgery, and arthroplasty, in which Level III and IV studies dominate citation rankings.^1^^,^^8^^,^^19^^,^^27^ The predominance of mid-level evidence likely reflects the practical and surgical nature of the specialty; many influential studies introduce new operative techniques, evaluate implant performance, or report outcomes of procedures that do not readily lend themselves to randomized designs due to ethical, logistical, or preference-based constraints.^1^^,^^8^^,^^19^^,^^27^ As a result, citation impact in shoulder and elbow surgery appears to be driven more by clinical relevance, technical innovation, and early descriptive outcomes than by methodological hierarchy. The scarcity of high-level comparative trials limits the strength of evidence underpinning clinical guidelines and emphasizes the need for multicenter collaboration, prospective study designs, and improved methodological consistency to support more robust evidence-based practice in shoulder and elbow surgery.

Authorship analyses typically emphasize first or corresponding author positions, potentially underrepresenting the influence of senior or collaborative authors.^21^ By examining first, last, and any author positions separately, this study reduces positional bias and provides a more comprehensive assessment of scholarly impact. Notably, the top 20 most-cited authors varied considerably across categories, underscoring the nuanced distribution of academic influence within the field.

Demographic trends in authorship reveal important insights into the evolving landscape of shoulder and elbow research. Several recent investigations in other surgical specialties have highlighted increasing contributions from private practice clinicians, women, and international collaborators.^14^^,^^17^ Consistent with these findings, our analysis found that 50.8% of U.S.-based top-cited first authors practiced in private settings, while 49.2% were affiliated with academic institutions. This near-equal distribution suggests that impactful research in shoulder and elbow surgery is not restricted to large academic centers but is also driven by high-volume clinicians and specialized private practice groups. The United States accounted for the majority of top-cited authors, followed by France, Switzerland, Japan, and Germany—countries known to host high-volume shoulder and elbow centers. When stratified by gender, our analysis found that women were less well represented. However, this is likely due to women comprising a small proportion of the shoulder and elbow surgeon workforce.^33^ This is reflected by organizations, such as the American Shoulder and Elbow Surgeons where women comprise 6.7% of their membership base.^33^

Recent work has demonstrated a steady increase in the number of authors per manuscript.^24^ This is in accordance with our own findings, as between *JSES*’ inception and the present, the number of authors per manuscript has more than doubled. This trend combined with existing literature revealing that the proportion of first authors with non-medical graduate degrees has increased suggests broader interdisciplinary collaboration and the growing involvement of research scientists, engineers, and statisticians in musculoskeletal research.^24^ At the same time, the proportion of clinical studies presenting original data has declined, while systematic reviews, meta-analyses, and narrative reviews have become more common.^23^^,^^24^ This development implies a maturation of the field. One in which researchers increasingly synthesize existing evidence but face ongoing challenges in conducting large-scale, high-quality prospective trials. Although longitudinal changes in the proportion of original clinical studies versus review articles were not directly analyzed within the present dataset and beyond the scope of this citation-focused analysis, this trend is noteworthy and warrants future investigation.

Overall, this study provides insight into the evolution of scholarly influence within shoulder and elbow research and highlights several opportunities for future advancement. Increasing the production of high-level comparative studies and promoting collaborative multinational research networks will be critical for enhancing the scientific contributions and clinical impact of *JSES* in the decades ahead.

### Limitations

This study has several limitations inherent to citation-based analyses. First, since citation data was extracted from Scopus at a single time point, articles assigned with future publication years through early indexing were included. Additionally, citation counts are influenced by numerous external factors—including journal visibility, self-citation practices, and the “snowball effect,” in which already highly cited papers continue to attract disproportionate attention, making citations an imperfect proxy for scientific quality.^13^^,^^21^

Second, this analysis was restricted entirely to *JSES* and therefore does not capture influential upper-extremity research published in other high-impact journals, potentially underestimating the contributions of authors or regions more active outside *JSES*.

Third, although demographic characteristics were extracted from publicly available sources, data on race, ethnicity, academic rank, and additional aspects of diversity were not consistently available, limiting a deeper analysis of representation. Prior bibliometric analyses have similarly lacked disaggregated data on authorship diversity, making it difficult to determine trends over time or assess progress toward greater inclusivity.^24^ Future research should incorporate more comprehensive demographic analyses to better understand representation and equity within upper-extremity research. Fourth, bibliometric methods cannot account for the clinical or educational impact of individual studies, nor do they distinguish between positive and negative citations.

Finally, assigning evidence levels retrospectively may introduce classification bias, and the use of Scopus as the sole database may result in differences from Web of Science or Google Scholar citation counts. These limitations should be considered when interpreting the findings, and future bibliometric studies may benefit from cross-database comparisons and expanded author-level demographic analyses.

## Conclusion

This comprehensive bibliometric evaluation demonstrates that highly cited *JSES* publications are predominantly clinical studies with mid-level (II–IV) evidence, reflecting both the practical nature of shoulder and elbow surgery and challenges in conducting randomized trials. Authorship within *JSES* can be mostly attributed to those from North America and Western Europe. Notably, private-practice clinicians contributed nearly half of the most influential publications, underscoring the broad base of expertise within the field. Together, these findings highlight the current trends within the journal as well as key opportunities for advancing shoulder and elbow research, including increasing the production of high-level comparative studies and promoting collaborative research networks. Strengthening methodological rigor within *JSES* publications will further enhance the journal's scientific impact and ensure continued progress in upper-extremity surgery.

## Disclaimers:

Funding: No funding was disclosed by the authors.

Conflicts of interest: The authors, their immediate families, and any research foundations with which they are affiliated have not received any financial payments or other benefits from any commercial entity related to the subject of this article.

## Supplementary Data

Supp Table 1

Supp Table 2

Supp Table 3
