# Supplementary material for: Bibliometric analysis of the Journal of Shoulder and Elbow Surgery: citation trends, evidence levels, and scholarly impact
Source: JSES Int. 2026 Mar 6;10(4):101691. doi: 10.1016/j.jseint.2026.101691 (PMC13158399; doi:10.1016/j.jseint.2026.101691)
Supplement: Supp Table 1 [file mmc1.pdf]

Supp Table 1. Top 100 Authors in the *Journal of Shoulder and Elbow Surgery* Ranked by Total Citations as an Author in Any Position

Rank	Authors	Citations	Articles	RCI
1	G., Walch, Gilles	10908	95	114.8
2	C.A., Gerber, Christian A.	8680	81	107.2
3	R.H., Cofield, Robert H.	8412	122	69
4	P., Boileau, Pascal	7762	66	117.6
5	J.W., Sperling, John W.	7191	134	53.7
6	J.D., Zuckerman, Joseph D.	7140	96	74.4
7	J.P., Iannotti, Joseph P.	6503	73	89.1
8	R.F., Warren, Russell Frederick	5643	78	72.3
9	L.U., Bigliani, Louis U.	5280	45	117.3
10	G.R., Williams, Gerald Ross	4726	71	66.6
11	M.A., Frankle, Mark A.	4327	77	56.2
12	J.J.P., Warner, Jon J.P.	4285	64	67
13	A.A., Romeo, Anthony A.	4257	86	49.5
14	E.L., Flatow, Evan L.	3748	48	78.1
15	T.B., Edwards, Thomas Bradley	3717	48	77.4
16	F.A., Matsen Iv, Frederick A.	3674	80	45.9
17	T.W., Wright, Thomas W.	3645	101	36.1
18	G.M., Gartsman, Gary M.	3643	27	134.9
19	K., An, Kainan	3354	48	69.9
20	J.L., Sánchez-Sotelo, Joaquín L.	3095	94	32.9
21	T.Q., Lee, Thay Q.	3092	63	49.1
22	R.J., Hawkins, Richard J.	3033	42	72.2
23	G.P., Nicholson, Gregory P.	3019	68	44.4
24	G.S., Athwal, George S.	2954	98	30.1
25	R.Z., Tashjian, Robert Zaray	2916	62	47
26	E., Itoi, Eijii	2892	50	57.8
27	R.J., Friedman, Richard Joel	2661	42	63.4
28	S.W., O'Driscoll, Shawn W.M.	2631	71	37.1
29	M.L., Ramsey, Matthew Lee	2628	42	62.6

30	J.A., Sidles, John A.	2539	13	195.3
31	S., Namdari, Surena	2478	76	32.6
32	B.F., Morrey, Bernard F.	2461	55	44.7
33	J.A., Johnson, James A.	2430	68	35.7
34	C.S., Ahmad, Christopher S.	2403	33	72.8
35	L.V., Gulotta, Lawrence Vincent	2386	45	53
36	B.J., Cole, Brian J.	2369	42	56.4
37	W.N., Levine, William N.	2329	47	49.6
38	D.M., Dines, David M.	2309	50	46.2
39	N.N., Verma, Nikhil N.	2273	51	44.6
40	R.R., Richards, Robin R.	2269	10	226.9
41	C.D., Schleck, Cathy D.	2265	29	78.1
42	C.P., Roche, Christopher P.	2255	45	50.1
43	R.W., Hertel, Ralph W.	2218	22	100.8
44	K., Yamaguchi, Ken	2213	22	100.6
45	G.J.W., King, Gregory J.W.	2197	62	35.4
46	L.J., Soslowsky, Louis J.	2164	24	90.2
47	E.R., Wagner, Eric R.	2138	36	59.4
48	J.C., Levy, Jonathan Chad	2132	54	39.5
49	J.A., Abboud, Joseph Albert	2089	70	29.8
50	E.V., Craig, Edward V.	2056	25	82.2
51	L., Neyton, Lionel	2051	25	82
52	P.J., Denard, Patrick Joel	2039	53	38.5
53	P.N., Chalmers, Peter Nissen	2021	67	30.2
54	J.E., Tibone, James Eugene	2002	29	69
55	D.W., Altchek, David W.	1999	24	83.3
56	K.J., Faber, Kenneth John	1983	41	48.4
57	V.C., Mow, Van C.	1982	5	396.4
58	L., Nové-Josserand, Laurent	1953	18	108.5
59	M.H., McGarry, Michelle H.	1943	51	38.1
60	J.D., Keener, Jay D.	1861	41	45.4
61	S.P., Steinmann, Scott P.	1839	35	52.5

62	F.W., Jobe, Frank W.	1816	14	129.7
63	P.H., Flurin, Pierre Henri	1815	28	64.8
64	M.M., Pink, Marilyn M.	1814	15	120.9
65	L.A., Michener, Lori Ann	1765	8	220.6
66	B.S., Olsen, Bo Sanderhoff	1743	33	52.8
67	J.Y., Bishop, Julie Y.	1726	29	59.5
68	L.M., Galatz, Leesa M.	1663	28	59.4
69	D., Molé, Daniel	1652	9	183.6
70	P.W., McClure, Philip W.	1604	6	267.3
71	S., Gumina, Stefano	1599	18	88.8
72	L.K., Hovelius, Lennart K.	1582	8	197.8
73	J.O., Söjbjerg, Jens Ole	1568	23	68.2
74	J.E., Carpenter, James E.	1556	15	103.7
75	A., Lädermann, Alexandre	1555	27	57.6
76	P., Habermeyer, Peter	1555	18	86.4
77	B., Mélis, Barbara	1540	10	154
78	D.R., Pupello, Derek R.	1539	13	118.4
79	C., Lévigne, Christophe;	1537	9	170.8
80	B.T., Feeley, Brian Thomas	1528	37	41.3
81	B.J., Sennett, Brian J.	1528	3	509.3
82	J.E., Kuhn, John E.	1509	23	65.6
83	L.D.B., Higgins, Laurence De Bree	1503	33	45.5
84	D.C., Ring, David C.	1502	41	36.6
85	O., Levy, Ofer	1497	33	45.4
86	D.C.M., Meyer, Dominik Christoph M.	1491	27	55.2
87	N., Yamamoto, Nobuyuki	1490	33	45.2
88	J.S., Dines, Joshua S.	1487	43	34.6
89	H., Minagawa, Hiroshi	1486	13	114.3
90	B., Jost, Bernhard	1478	22	67.2
91	H., Resch, Herbert	1478	24	61.6
92	A.S., Rokito, Andrew S.	1475	20	73.8

93	T.R., Norris, Tom R.	1447	15	96.5
94	C.M., Rowland, Charles M.	1444	8	180.5
95	A., Gristina, Anthony	1441	1	1441
96	S.G.B., Krishnan, Sumant G.Butch	1438	12	119.8
97	N.S., ElAttrache, Neal S.	1437	18	79.8
98	Y.W., Kwon, Young W.	1432	22	65.1
99	F.A., Cordasco, Frank A.	1391	23	60.5
100	L., Favard, Luc	1383	13	106.4
