# Supplementary material for: Bibliometric analysis of the Journal of Shoulder and Elbow Surgery: citation trends, evidence levels, and scholarly impact
Source: JSES Int. 2026 Mar 6;10(4):101691. doi: 10.1016/j.jseint.2026.101691 (PMC13158399; doi:10.1016/j.jseint.2026.101691)
Supplement: Supp Table 2 [file mmc2.pdf]

Supp Table 2. Top 100 Authors in the *Journal of Shoulder and Elbow Surgery* Ranked by Total Citations as the First Author

**Rank**	**Author**	**Total First Citations**	**First Author Count**	**First Author RCI**	**First Author RCI Ranking**	**Total Last Author Citations**	**Last Author Count**	**Last Author RCI**
1	P., Boileau, Pascal	4127	24	172	35	1005	20	50.3
2	G., Walch, Gilles	2869	12	239.1	22	5261	47	M
3	R.Z., Tashjian, Robert Zaray	1571	19	82.7	63	664	18	36.9
4	R.R., Richards, Robin R.	1517	3	505.7	5	466	4	116.5
5	L.K., Hovelius, Lennart K.	1512	7	216	26	-	-	-
6	C.A., Gerber, Christian A.	1378	11	125.3	46	6548	54	121.3
7	R.W., Hertel, Ralph W.	1341	8	167.6	36	616	9	68.4
8	B., Fuchs, Bruno	1323	2	661.5	3	-	-	-
9	J.W., Sperling, John W.	1202	10	120.2	48	857	24	35.7
10	A., Yamamoto, Atsushi	1175	3	391.7	12	-	-	-
11	J.J.P., Warner, Jon J.P.	1042	12	86.8	62	1994	33	60.4
12	T.B., Edwards, Thomas Bradley	1016	8	127	45	1234	21	58.8
13	L.A., Michener, Lori Ann	979	2	489.5	6	95	3	31.7
14	N., Yamamoto, Nobuyuki	940	9	104.4	52	-	-	-
15	D., Goutallier, Daniel	932	3	310.7	15	18	2	9

16	J.S., Day, Judd S.	896	6	149.3	40	-	-	-
17	G.M., Gartsman, Gary M.	895	9	99.4	57	321	6	53.5
18	R.W., Nyffeler, Richard W.	884	4	221	25	-	-	-
19	O., Levy, Ofer	867	12	72.3	77	203	9	22.6
20	F., Postacchini, Franco	862	2	431	8	335	6	55.8
21	L.J., Soslowsky, Louis J.	858	3	286	17	980	15	65.3
22	J., Oh, Joo-han	836	16	52.3	92	180	14	12.9
23	P.N., Chalmers, Peter Nissen	776	19	40.8	98	151	11	13.7
24	M.C., Park, Maxwell C.	763	4	190.8	32	-	-	-
25	B.T., Elhassan, Bassem T.	759	12	63.3	87	214	9	23.8
26	K., Yamaguchi, Ken	756	4	189	34	720	8	90
27	S., Namdari, Surena	746	10	74.6	75	1157	44	26.3
28	M.D., McKee, Michael David	733	3	244.3	20	292	6	48.7
29	S., Tempelhof, Siegbert	730	1	730	1	-	-	-
30	R.W., Simovitch, Ryan Wade	716	7	102.3	54	6	2	3
31	J.D., Zuckerman, Joseph D.	701	7	100.1	55	2309	40	57.25
32	P., Habermeyer, Peter	692	5	138.4	42	615	8	76.9
33	M.K., Gilbart, Michael K.	687	1	687	2	-	-	-
34	S., Antuña, Samuel	685	5	137	43	15	3	5
35	J., Nagels, Jochem	676	2	338	14	5	1	5

36	P., Olerud, Per	656	4	164	37	-	-	-
37	E.R., Wagner, Eric R.	638	8	79.8	65	418	11	38
38	J.E., Kuhn, John E.	631	3	210.3	27	513	7	73.3
39	A.D., Armstrong, April Dawn	626	6	104.3	53	523	10	52.3
40	P.G., Collin, Philippe G.	621	8	77.6	70	155	7	22.1
41	J.P., Iannotti, Joseph P.	620	8	77.5	71	2955	38	77.8
42	M.E., Torchia, Michael E.	609	2	304.5	16	-	-	-
43	L., Neyton, Lionel	602	9	66.9	81	2	1	2
44	B.C., Werner, Brian Christopher	592	15	39.5	99	248	13	19
45	S., Gutiérrez, Sergio	573	3	191	31	-	-	-
46	A., Lädermann, Alexandre	565	7	80.7	64	184	6	30.7
47	P.W., McClure, Philip W.	553	1	553	4	72	3	24
48	J.Y., Bishop, Julie Y.	552	2	276	18	204	3	68
49	R.S., Churchill, R. Sean	550	7	78.6	68	49	3	16.3
50	S.J., Nho, Shane Jay	549	6	91.5	59	-	-	-
51	E.T., Ek, Eugene T.	537	4	134.3	44	40	5	8
52	G.P., Nicholson, Gregory P.	534	7	76.3	72	1045	15	69.7
53	J.C., Levy, Jonathan Chad	520	10	52	93	548	30	18.3

54	T.R., Norris, Tom R.	512	2	256	19	544	6	90.7
55	A.A., Deutsch, Allen A.	498	5	99.6	56	110	1	110
56	Y.W., Kwon, Young W.	489	5	97.8	58	383	6	63.8
57	C., Cho, Chul-hyun	489	9	54.3	90	9	3	3
58	W.N., Levine, William N.	487	4	121.8	47	547	17	32.2
59	G.I., Groh, Gordon I.	481	8	60.1	88	43	1	43
60	S.B., Lippitt, Steven B.	479	2	239.5	21	56	1	56
61	J.D., Keener, Jay D.	478	7	68.3	80	602	17	35.4
62	W.W., Schairer, William W.	476	3	158.7	38	-	-	-
63	P.E., Mintken, Paul E.	474	1	474	7	-	-	-
64	B.J., Morris, Brent Joseph	474	6	79	66	-	-	-
65	C., Lévigne, Christophe	473	2	236.5	23	377	2	188.5
66	P., Clavert, Philippe	471	4	117.8	49	2	1	2
67	J., Ide, Junji	467	6	77.8	69	-	-	-
68	P.S., Randelli, Pietro S.	466	2	233	24	24	3	8
69	B., Jost, Bernhard	456	3	152	39	224	6	37.3
70	M.A., Frankle, Mark A.	452	4	113	50	2684	56	47.9
71	W.J., Mallon, William J.	452	6	75.3	74	200	8	25
72	S.J., Hattrup, Steven J.	451	11	41	96	80	4	20

73	J.G., Edelson, Jacob Gordon	451	11	41	97	266	5	53.2
74	E.V., Cheung, Emilie V.	450	7	64.3	86	127	5	25.4
75	F.A., Matsen Iv, Frederick A.	449	9	49.9	94	3123	52	60.1
76	B.S., Schoch, Bradley S.	449	15	29.9	100	273	21	13
77	B.S., Werner, Birgit S.	441	5	88.2	60	-	-	-
78	S.S., Hasan, Samer S.	440	4	110	51	17	2	8.5
79	B.S., Olsen, Bo Sanderhoff	436	5	87.2	61	191	7	27.3
80	P.J., Denard, Patrick Joel	431	6	71.8	78	319	14	22.8
81	E.M., Padegimas, Eric Michael	429	8	53.6	91	-	-	-
82	N.M., DiGiovine, Nick M.	422	1	422	9	14	1	14
83	J.A., Singh, Jasvinder A.	417	3	139	41	-	-	-
84	G.L., Cvetanovich, Gregory L.	412	6	68.7	79	31	3	10.3
85	A.V., Deshmukh, Ashwin V.	409	2	204.5	28	-	-	-
86	S.J., Snyder, Stephen J.	407	1	407	6,	240	4	60
87	M.J., Best, Matthew J.	405	2	202.5	29	16	2	8
88	C., Hand, Campbell	397	1	397	11	-	-	-
89	P., Mansat, Pierre	397	6	66.2	82	106	5	21.2

90	P.J., McMahon, Patrick J.	397	6	66.2	83	253	5	50.6
91	E., Itoi, Eijii	395	5	79	67	539	15	35.9
92	M.L., Pearl, Michael Lawrence	395	6	65.8	84	-	-	-
93	J.P., Kukkonen, Juha P.	390	2	195	30	-	-	-
94	D.J., Cuff, Derek J.	386	6	64.3	85	-	-	-
95	C.A., Cummins, Craig Anthony	381	2	190.5	33	-	-	-
96	C.P., Roche, Christopher P.	381	7	54.4	89	913	9	101.4
97	T.W.Q., Throckmorton, Thomas W.Quin	377	5	75.4	73	452	16	28.25
98	M.J., Bercik, Michael J.	376	1	376	13	-	-	-
99	H., Sano, Hirotaka	373	8	46.6	95	61	1	61
100	R.J., Friedman, Richard Joel	363	5	72.6	76	457	27	16.9
