# Supplementary material for: Bibliometric analysis of the Journal of Shoulder and Elbow Surgery: citation trends, evidence levels, and scholarly impact
Source: JSES Int. 2026 Mar 6;10(4):101691. doi: 10.1016/j.jseint.2026.101691 (PMC13158399; doi:10.1016/j.jseint.2026.101691)
Supplement: Supp Table 3 [file mmc3.pdf]

Supp Table 3. Top 100 Authors in the *Journal of Shoulder and Elbow Surgery* Ranked by Total Citations as the Last Author

Rank	Author	Total Last Author Citations	Last Author Count	Last Author RCI	First Author Rank
1	C.A., Gerber, Christian A.	6548	54	121.3	6
2	G., Walch, Gilles	5261	47	111.9	2
3	R.H., Cofield, Robert H.	4107	62	66.2	-
4	F.A., Matsen Iv, Frederick A.	3123	52	60.1	76
5	J.P., Iannotti, Joseph P.	2955	38	77.8	41
6	T.Q., Lee, Thay Q.	2839	51	55.7	-
7	M.A., Frankle, Mark A.	2684	56	47.9	71
8	J.D., Zuckerman, Joseph D.	2309	40	57.7	31
9	R.F., Warren, Russell Frederick	2175	27	80.6	-
10	E.L., Flatow, Evan L.	2010	23	87.4	-
11	J.J.P., Warner, Jon J.P.	1994	33	60.4	11
12	R.J., Hawkins, Richard J.	1734	22	78.8	-
13	L.U., Bigliani, Louis U.	1625	20	81.3	-
14	G.S., Athwal, George S.	1606	55	29.2	-
15	S.W., O'Driscoll, Shawn W.M.	1397	48	29.1	-
16	C.M., Rowland, Charles M.	1385	7	197.9	-
17	G.A.C., Murrell, G. A.C.	1292	28	46.1	-
18	T.B., Edwards, Thomas Bradley	1234	21	58.8	12
19	L.V., Gulotta, Lawrence Vincent	1167	21	55.6	-
20	S., Namdari, Surena	1157	44	26.3	27

21	A.A., Romeo, Anthony A.	1149	29	39.6	-
22	T., Kobayashi, Tsutomu	1057	2	528.5	-
23	G.P., Nicholson, Gregory P.	1045	15	69.7	52
24	D., Molé, Daniel	1032	4	258	-
25	G.J.W., King, Gregory J.W.	1027	31	33.1	-
26	O., Sneppen, Otto	1022	15	68.1	-
27	K., An, Kainan	1021	20	51.1	-
28	C.S., Ahmad, Christopher S.	1007	17	59.2	-
29	P., Boileau, Pascal	1005	20	50.3	1
30	L.J., Soslowsky, Louis J.	980	15	65.3	21
31	I., Hovorka, Istvàn	957	1	957	-
32	J.A., Johnson, James A.	941	20	47.1	-
33	J.L., Sánchez-Sotelo, Joaquín L.	922	31	29.7	-
34	C.P., Roche, Christopher P.	913	9	101.4	95
35	B.J., Sennett, Brian J.	904	1	904	-
36	J.W., Sperling, John W.	857	24	35.7	9
37	S.A., Copeland, Stephen A.	841	15	56.1	-
38	G.R., Williams, Gerald Ross	835	14	59.6	-
39	P.M., Rozing, Pieter M.	829	7	118.4	-
40	S., Van-Driessche, Stéphane	814	1	814	-
41	D.C., Ring, David C.	808	22	36.7	-
42	B.F., Morrey, Bernard F.	804	23	35	-
43	C.A., Rockwood, Charles A.	790	14	56.4	-
44	A.J., Carr, Andrew Jonathan	779	12	64.9	-

45	A.S., Rokito, Andrew S.	776	6	129.3	-
46	S.M., Kurtz, Steven M.	774	3	258	-
47	K., Okada, Kyoji	746	5	149.2	-
48	T.W., Wright, Thomas W.	736	18	40.9	-
49	R., Seil, Romain	730	1	730	-
50	K., Yamaguchi, Ken	720	8	90	26
51	D.E., Eygendaal, Denise E.	713	22	32.4	-
52	F., Albo, Francesco	706	1	706	-
53	F.E., Gohlke, Frank Erol	690	11	62.7	-
54	N.N., Verma, Nikhil N.	665	12	55.4	-
55	R.Z., Tashjian, Robert Zaray	664	18	36.9	3
56	S.P., Steinmann, Scott P.	661	11	60.1	-
57	A.M., Chamberlain, Aaron M.	658	7	94	-
58	J., Perry, Jacqueline	656	4	164	-
59	J.M., Wiater, Jerome Michael	638	18	35.4	-
60	P.J., Millett, Peter J.	630	18	35	-
61	R.J.H., Emery, Roger J.H.	626	10	62.6	-
62	M., Leunig, Michael	625	1	625	-
63	R.W., Hertel, Ralph W.	616	9	68.4	7
64	P., Habermeyer, Peter	615	8	76.9	32
65	S.T., Donell, Simon T.	610	1	610	-
66	M.B., Gottschalk, Michael B.	605	6	100.8	-
67	J., Tidermark, Jan	605	3	201.7	-
68	W.E., Lee, William E.	603	4	150.8	-
69	J.D., Keener, Jay D.	602	17	35.4	61
70	B.T., Feeley, Brian Thomas	601	12	50.1	-

71	C.R., Settergren, Curtis R.	595	1	595	-
72	W.J., Willems, Willem Jaap	586	10	58.6	-
73	A.R., Karduna, Andrew R.	586	2	293	-
74	S.F., Brockmeier, Stephen F.	577	10	57.7	-
75	A., Green, Andrew	575	12	47.9	-
76	I., Szabó, Istvan	555	1	555	-
77	A.M., Farron, Alain M.	552	12	46	-
78	A.P., Powell, Amy P.	549	1	549	-
79	J.C., Levy, Jonathan Chad	548	30	18.3	53
80	W.N., Levine, William N.	547	17	32.2	58
81	T.R., Norris, Tom R.	544	6	90.7	54
82	U., Srikumaran, Uma	542	18	30.1	-
83	C.B., Ma, Chunbong Benjamin	542	7	77.4	-
84	E., Itoi, Eijii	539	15	35.9	92
85	D.D., D'Lima, Darryl D.	535	9	59.4	-
86	F.W., Jobe, Frank W.	532	5	106.4	-
87	A.D., Armstrong, April Dawn	523	10	52.3	39
88	V.O., Äärimaa, Ville O.	514	6	85.7	-
89	J.E., Kuhn, John E.	513	7	73.3	38
90	D.P., O'Connor, Daniel Patrick	512	3	170.7	-
91	M., Saebö, Modolv	512	2	256	-
92	J.D., MacGillivray, John D.	511	5	102.2	-
93	H., Resch, Herbert	498	11	45.3	-
94	S.A., Rodeo, Scott Alan	492	6	82	-

95	W.D., Middleton, William D.	487	1	487	-
96	C.J., Basamania, Carl J.	479	4	119.8	-
97	S.A., Fealy, Stephen A.	476	6	79.3	-
98	J.A., Cleland, Joshua Aland	474	1	474	-
99	F.H., Fu, Freddie H.	468	2	234	-
100	R.R., Richards, Robin R.	466	4	116.5	4
